# Aflibercept for treatment-naïve diabetic macula oedema in a multi-ethnic population: Real-world outcomes from North West London

**DOI:** 10.1371/journal.pone.0246626

**Published:** 2021-02-11

**Authors:** Christiana Dinah, Arevik Ghulakhszian, Sing Yue Sim, Amal Minocha, Soroush Nokhostin, Esther Posner, Richard Cheong-Lee, Sheena George

**Affiliations:** 1 Department of Ophthalmology, London North West University Healthcare NHS Trust, London, United Kingdom; 2 Hillingdon Hospital NHS Foundation Trust, London, United Kingdom; 3 Imperial College London, London, United Kingdom; 4 Western Eye Hospital, Imperial College Healthcare NHS Trust, London, United Kingdom; University of Warmia, POLAND

## Abstract

**Purpose:**

To evaluate the clinical outcomes of patients with treatment–naïve diabetic macula oedema (DMO) treated with Aflibercept in routine clinic settings in ethnically diverse North West London (NWL) and compare to outcomes reported in the VIVID and VISTA clinical trials

**Methods:**

This was a retrospective multicentre interventional case series. Two hundred and seventy eyes of 221 treatment-naïve patients at three NWL hospitals initiated on Aflibercept and who had at least 12 months follow-up were included in the study. Visual acuity, central subfield thickness and macula volume were recorded at baseline, month 3, 6 and 12.

**Results:**

There were significant differences between the NWL cohort and participants in the VIVID and VISTA trials at baseline including higher HbA1c and a higher proportion of eyes with proliferative diabetic retinopathy in the NWL cohort. The mean VA, mean CSFT and mean MV at baseline was 66.4 (± 14.6) letters, 417 (± 94) μm and 10.3 (± 1.9) mm^3^. The mean VA gain at 12 months was 4.0 (± 11.8) letters (p < 0.001); a total of 26% of eyes gained ≥ 10 letters, 15% of eyes gained ≥ 15 letters and 6% lost ≥15 letters. At 12-months, the mean reduction in CSFT was 108 (± 96) μm (p<0.001) and the mean reduction in MV was 1.05 (± 1.21) mm^3^ (p<0.001). An average of 6.2 (± 2.3) injections was given over 12 months. There was a significant association between functional and anatomical response category at 3 months and response category at 12 months (p<0.001).

**Conclusion:**

The effectiveness of treatment with Aflibercept for patients in NWL was meaningfully lower than was reported in the VIVID and VISTA clinical trials. A high proportion of patients with good visual acuity at baseline, poorer glycaemic control, worse diabetic retinopathy and under-treatment likely contributed to lower functional and anatomical outcomes.

## Introduction

Diabetic macula oedema (DMO) is a major cause of visual loss in patients with diabetes [1–3]. 3.9 million people in the United Kingdom are currently diagnosed with diabetes with the estimated prevalence predicted to rise to 5.3 million by 2025 [4].

North West London (NWL) is a region of the London comprising 8 boroughs, 3 of which are the most ethnically diverse in the country. In Brent, 63.7% of residents are non-Caucasian, with 57.8% and 51% of residents identifying as non-Caucasian in Harrow and Ealing respectively [5]. The prevalence of diabetes in this region is also amongst the highest in the country, with 9.58% of Harrow residents diagnosed with diabetes and 8.91% and 8.24% in Brent and Ealing respectively compared to the London average of 6.5% [6].

Currently, there are 2 vascular endothelial growth factor (VEGF) inhibitors, Ranibizumab and Aflibercept, are approved for treatment of centre-involving diabetic macula oedema in the United Kingdom. Ranibizumab received National Institute of Health and Care Excellence (NICE) approval for DMO in 2013 [7], followed by Aflibercept in 2015 [8]. Aflibercept is a soluble decoy receptor fusion protein that inhibits placental growth factor (PIGF) in addition to VEGF-A and VEGF-B. It has a 100-fold greater binding affinity for VEGF-A than intravitreal ranibizumab [9], which provides the potential for less frequent dosing with substantial savings in cost and treatment burden to patients. The approval of Aflibercept by NICE was largely based on evidence from the landmark VISTA and VIVID clinical trials [10], in which 30–40% of patients gained ≥15 letters although the studies did not include sites in the United Kingdom. Additional evidence for the efficacy of Aflibercept was provided in the DRCR.net Protocol T trial [11], which showed Aflibercept to be superior to ranibizumab and bevacizumab for DMO, particularly in the subgroup of patients with visual acuity ≤69 letters. In all eye departments in NWL, Aflibercept is the first line treatment of choice for centre-involving macula oedema. However, the study population in the landmark clinical trials are demographically different from those that routinely attend eye departments in North West London and clinical trials by definition do not take into account poor glycaemic control, multiple comorbidities, missed or delayed appointments and the influence of under treatment on functional and visual outcomes.

The goal of the present study was to evaluate the clinical outcomes of patients with treatment-naïve diabetic macula oedema treated with Aflibercept in routine clinic settings in NWL and compare them to outcomes reported in the VISTA and VIVID study.

## Methods

This was a retrospective, multicentre cohort study of treatment naïve diabetic macular oedema patients initiated on intravitreal Aflibercept at three North West London hospitals between January 2016 and July 2018. The study was approved prospectively by the Research and Development departments of all 3 hospitals (London North West University Healthcare NHS Trust Research and Development reference no: SE19/016, Imperial College Healthcare NHS Trust service evaluation reference no: 381 and Hillingdon Hospital NHS Foundation Trust service evaluation reference no: 1018) and the study followed the tenets of the declaration of Helsinki.

Patients initiated on intravitreal Aflibercept for centre-involving diabetic macula oedema with at least 12 months follow-up were enrolled. There was no visual acuity threshold for initiation of treatment at the time in NWL and the central subfield thickness (CSFT) at initiation could be less than <400μm if there was an area greater than 400μm in the central 3000μm of the Early Treatment Diabetic Retinopathy Study (ETDRS) grid. The exclusion criteria were cataract surgery within 3 months of commencing intravitreal Aflibercept, other macula disease such as retinal vein occlusion or age-related macula degeneration and presence of macula oedema of other aetiology. Patients with all grades of diabetic retinopathy, previous macula laser or panretinal laser, vitreoretinal interface disturbance that did not require surgical intervention where included. We included these patients because they reflect our routine practice where the full range of diabetic macula oedema is treated. The treatment protocol in all 3 hospitals was in alignment with 2014 EU summary of product characteristics label for Aflibercept which is loading phase of 5 monthly intravitreal Aflibercept (unless success after 3 injections), followed by ongoing injections to stabilise visual acuity or treat residual oedema if required and typically at 8 weekly intervals. For patients who received bilateral treatment, both eyes were included.

### Data collection

Electronic medical records for patients were reviewed for demographic data, HbA1c values (within 6 months of first injection), creatinine and eGFR (within 6 months of first injection), retinopathy status using the United Kingdom national screening committee (NSC) grades (R1: mild and moderate non-proliferative diabetic retinopathy, R2: severe non-proliferative diabetic retinopathy, R3A: active proliferative diabetic retinopathy, R3S: stable treated proliferative diabetic retinopathy) as documented in the medical records at baseline, total number of injections at 6 and 12 months and best corrected visual acuity (BCVA) at baseline, 3 months, 6 months and 12 months. Visual acuity was assessed using ETDRS charts at 4 metres at baseline and at all injection visits. OCT scans were obtained using SD OCT: Heidelberg Spectralis, Heidelberg, Germany at London North West University Healthcare NHS Trust and Imperial College NHS Trust and HD OCT Cirrus 5000 (Carl Zeiss AG, Germany) at Hillingdon Hospital NHS Foundation Trust. Quantitative assessment of DMO was performed at all 3 sites and included central subfield thickness CSFT and macula volume, which were calculated automatically by the instrument and recorded at baseline and at 3, 6 and 12 months after the first intravitreal Aflibercept. The efficacy endpoint documented was the CSFT defined as the thickness of the central 1000μm on the ETDRS grid. Qualitative evaluation of OCT images was performed for OCT images of patients attending London North West University Healthcare NHS Trust and Hillingdon Hospital NHS Foundation Trust only (212 eyes) and that will be presented in a further paper on prognostic factors influencing functional and anatomical outcomes. All data was collected at scheduled time points ± 1 month to allow for scheduling and capacity issues within each hospital.

### Outcome measures

Our primary outcome measure was a change from baseline BCVA in ETDRS letters at 12 months. Our secondary outcome measures included a) the proportion of eyes that gained ≥ 10 letters from baseline b) proportion of eyes that gained ≥15 letters from baseline c) proportion that lost ≥15 letters from baseline, d) change from baseline in central macula thickness and macula volume and e) proportion of intraocular inflammation and infectious endophthalmitis. Exploratory objectives were to assess whether response at 3 months was predictive of response at 12 months.

### Statistical methods

Sample measurements have been summarised with mean value and standard deviation. Comparison between baseline and final parameters were done by paired t-test. Chi-squared test was used to evaluate the association between outcomes at 3 months and 12 months. Data collection and analyses were done using Microsoft Excel 2010 (Microsoft Corporation, Washington, USA) and SPSS 22.0 (SPSS Inc., Chicago, USA). A p value of <0.05 was interpreted as statistically significant.

## Results

### Baseline demographics

A total of 221 patients (270 eyes) met the inclusion criteria for the study. Baseline demographics are presented in Table 1. There was, as expected, significant ethnic diversity with 35.3% of patients of South Asian origin, 28% Caucasian, 21.7% Black or Afro-Caribbean and 5% Far East Asian. 22.9% of eyes were categorised as R1, 55.6% as R2, 15.9% as R3A and 5.2% as R3S. The mean HbA1c for our patient group was 8.3 ± 4. The mean number of injections during the 12 month period was 6.2 ± 2.3.

**Table 1:** Patient demographics and baseline characteristics and comparison to VIVID and VISTA participants.

Characteristic	NWL	VISTA	VIVID
	(patient, n = 221 eyes, n = 270)	IAI 2q8 (eyes, n = 151)	IAI 2q8 (eyes, n = 135)
Mean age, years (SD)	62.8 (12.6)	63.1 (9.4)	64.2 (7.8)
Sex, n (%)			
Male	147 (66.5)	78 (51.7)	88 (65.2)
Female	74 (33.5)	73 (48.3)	47 (34.8)
Ethnicity, n (%)			
Black	48 (21.7)	19 (12.6)	1 (0.7)
White	62 (28.0)	125 (82.8)	106 (78.5)
Far East Asian	11 (5.0)	2 (1.3) **	27 (20.0) **
South Asian	78 (35.3)		
Other	-	5 (3.3)	1 (0.7)
Unknown	22 (10.0)	-	-
Mean HbA1c, % (SD)	8.3 (4)	7.9 (1.6)	7.7 (1.4)
Mean eGFR	72	-	-
Mean creatinine	88	-	-
Retinopathy status, n (%)			
R0	0 (0)	4 (2.6)	0 (0)
R1	62 (22.9)	64 (42.4)	29 (21.4)
R2	150 (55.6)	72 (47.7)	69 (51.1)
R3A	43 (15.9)	8 (5.3)	3 (2.2)
R3S	14 (5.2)	-	-
Ungradable	-	3 (2.0)	34 (25.2)
Lens status, n (%)			
Phakic	208 (77.0)	-	-
Pseudophakic	51 (18.9)	-	-
Mean BCVA, letters (SD)	66.4 (14.6)	59.4 (10.9)	58.8 (11.2)
Mean central retinal thickness, μm (SD)	418 (94.0)	479 (154)	518 (147)
Mean injections, n (SD)	6.2 (2.3)	8.4 (1.3)	8.7 (1.2)

*NWL-* North West London data set; *IAI 2q8* –Cohort of patients receiving 2mg intravitreal aflibercept (IAI) every 4 weeks from baseline to week 16 (5 doses) followed by dosing every 8 weeks through week 48; *SD–*Standard Deviation; *HbA1c-* Glycated haemoglobin; *eGFR–*Estimated Glomerular Filtration Rate; *R0*- No retinopathy; *R1*- Background retinopathy; *R2*- Pre-proliferative retinopathy; *R3A*- Active proliferative retinopathy; *R3S*- Stable proliferative retinopathy; *BCVA-* Best Corrected Visual Acuity.

**In VIVID and VISTA studies [18] “Asian” subgroups were not differentiated.

### Functional outcomes

**Fig 1:**
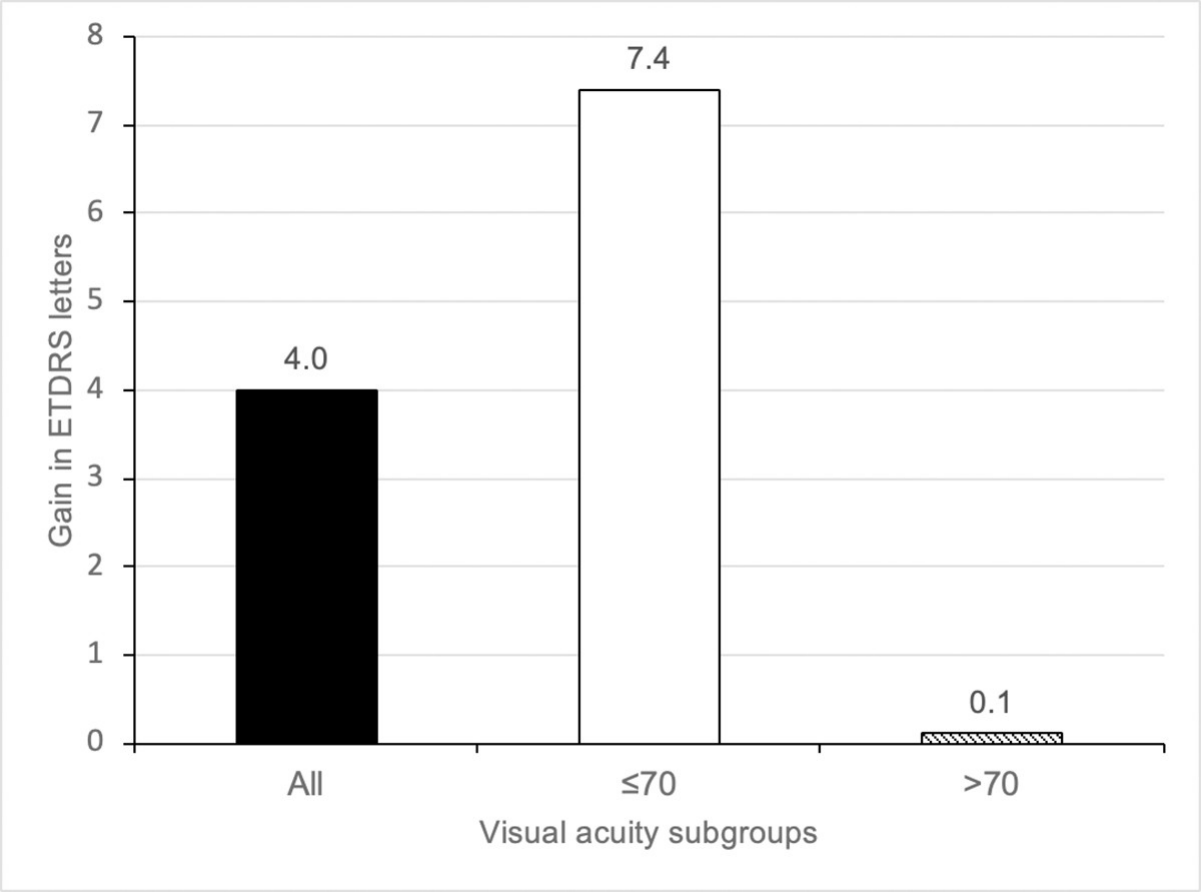
Visual acuity gain at 12 months by baseline visual acuity subgroup.

**Fig 2:**
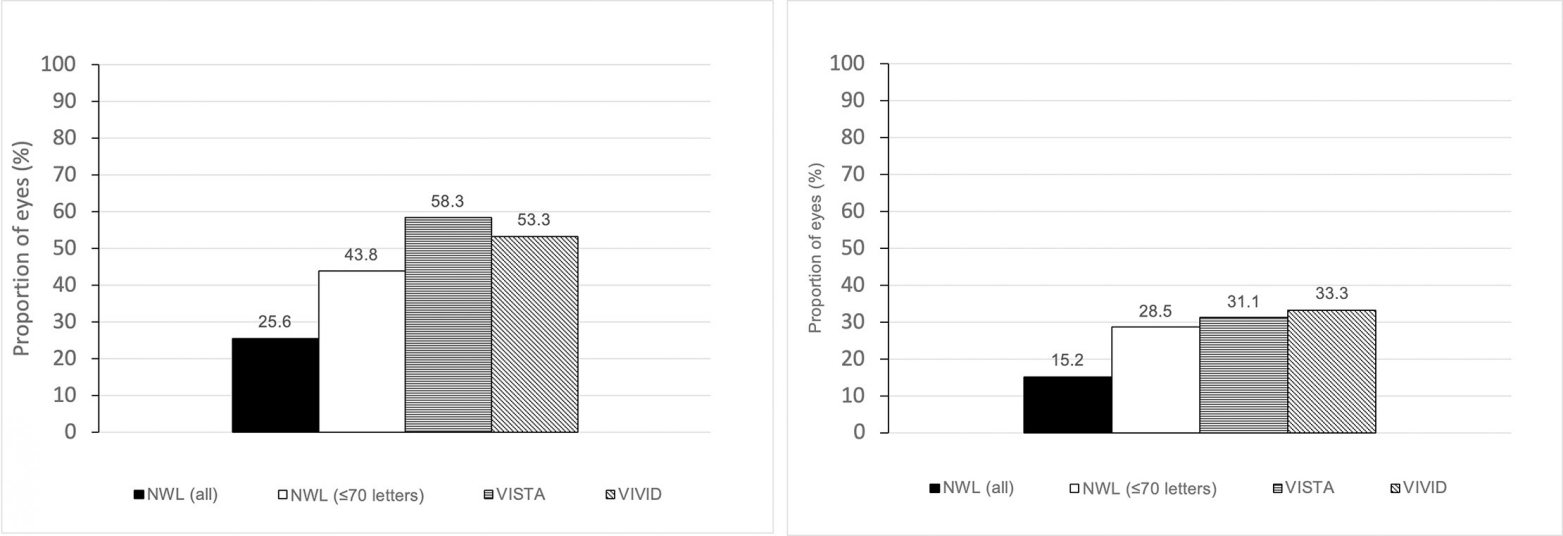
A. Proportion of eyes with ≥10 letters gained at 12 months in the NWL cohort and the VIVID and VISTA trials. B. Proportion of eyes with ≥15 letters gained at 12 months in the NWL cohort and the VIVID and VISTA trials.

The mean BCVA at baseline was 66.4 ± 14.6 ETDRS letters. The mean gain in BCVA was 4.0 ± 11.8 (p < 0.001) at 12-months (Fig 1). 26% and 15% of eyes improved by ≥10 and ≥15 letters respectively at 12 months and 6% of eyes lost ≥15 letters (Fig 2A and 2B). Patients were also sub-divided into three groups based on their functional response; limited (<5 letters gain), moderate (5–9 letters) and good (10+ letters) visual gain. At 12-months, 55.0% of eyes demonstrated limited visual acuity gain, 19.0% and 26% demonstrated moderate and good gain respectively. Subgroup analysis of patients with baseline BCVA ≤70 letters (144 eyes, 53.3% of the total study population) was performed to address the possible effects of a ceiling on visual gain. These patients demonstrated a BCVA gain of 7.4 ± 13.9 (p <0.001) at 12-month (Fig 1). In this subgroup, at 12 months, 38% had limited visual acuity gain, 18% and 44% demonstrated moderate and good visual acuity gain respectively, whilst 28.5% gained ≥15 letters (Fig 2A and 2B). In the subgroup of patients with VA >70, the mean visual gain was 0.1 ± 7 letters (p = 0.85). There was no incidence of infectious endophthalmitis and 2 patients (0.9%) had an episode of anterior uveitis which resolved with topical steroid treatment.

### Anatomical outcomes

Baseline mean CSFT was 417 ± 94. At 12 months, there was a mean reduction from baseline of 108 ± 96 which was significant (p<0.001). The mean macula volume at baseline was 10.3± 1.9 and the mean reduction in macula volume at 12 months was 1.05 ± 1.21 (p<0.001). The mean reduction in CSFT in the subgroup of patients with CSFT ≥400μm at baseline (160 eyes) was 142.3μm (p<0.001). The CSFT response was further classified into limited (<100μm reduction) and robust (≥100μm reduction) response. At 12 months, there were 135 eyes (52%) in the limited response category and 20% of the total cohort demonstrated CSFT reduction of ≤10%.

### Effects of loading treatment

Of 270 eyes included in the study, 45.5% (123 eyes) did not receive at least 5 intravitreal Eylea injections within 6 months. This was as a result of the patient not attending appointments in 43 eyes (35%), clinical capacity issues in 33 eyes (26.8%) and clinician’s judgement as a result of adequate response after 3 intravitreal injections in 47 eyes (41.5%). In summary, 76 eyes (28.1%) did not receive the complete loading dose as intended by the clinician due to patients not adhering to the treatment appointments or lack of clinical capacity. There was no significant difference in visual acuity gained at 12 months between those who completed the 5 loading doses and those who received less than 5 (p = 0.27).

### Predicting functional outcome by response at 3 months

**Fig 3:**
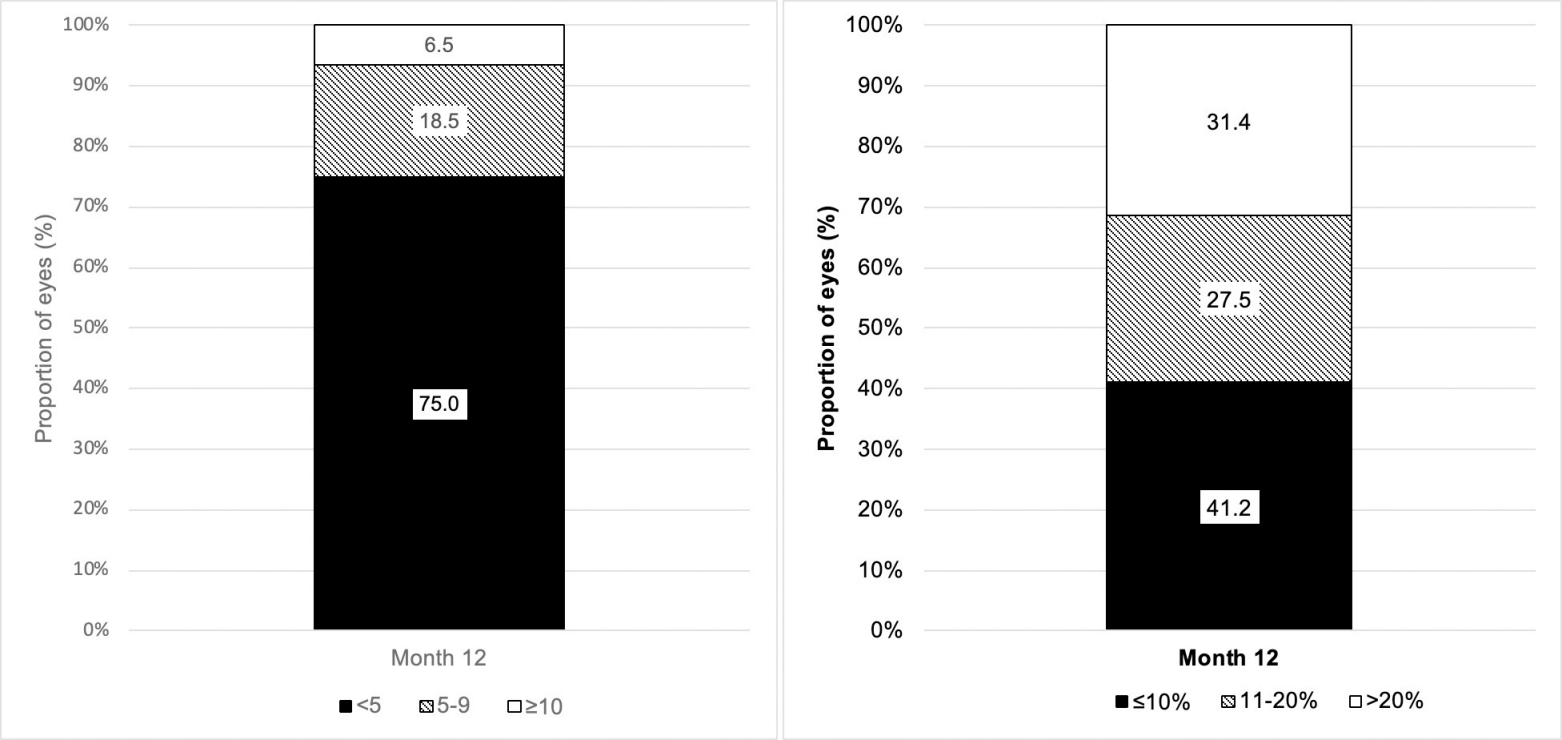
A. Visual acuity outcomes at 12 months in subgroup with limited visual acuity gain (<5 letters) at 3 months. B. Anatomic outcomes at 12 months in subgroup with limited response (≤10%) at 3 months.

We investigated the association between functional and anatomical response after 3 Aflibercept injections in eyes with visual acuity data at baseline, month 3 and month 12 (233 eyes). There was a significant association between functional and anatomical response category at 3 months and response category at 12 months (p<0.001). A limited visual acuity response did not entirely preclude later development of robust response; however this was in a minority of eyes. Within the subset of eyes with <5 letter gain at 3 months (124 eyes, 53.2%), only 23 eyes (18.5%) went on to gain between 5–9 letters at 12 months and 8 (6.5%) eyes subsequently gained >10 letters (Fig 3A). Similarly, in eyes with OCT data at all 3 time points (233 eyes) limited anatomical response at 3 months became robust in a moderate proportion of eyes by 12 months. Of the eyes with ≤10% reduction in CSFT, 31.4% demonstrated robust response at 12 months (Fig 3B).

## Discussion

The effectiveness of treatment with Aflibercept for patients in North West London was meaningfully lower than that reported in the registration trials. The visual acuity benefit observed in our cohort at 12 months was +4 letters compared to +10.7 in the 8 weekly arm of VISTA and VIVID. Similarly, 26% and 15% gained ≥10 letters and ≥15 letters in the NWL cohort compared to over 50% gaining ≥10 letters and over 30% gaining ≥15 letters in the VISTA and VIVID trials. 6% of our cohort also lost ≥15 letters compared to less than 1% in the clinical trials. This wide discrepancy can partly be explained by the fact that over 50% of patients in the NWL cohort had visual acuity >70 letters and it is recognised from multiple clinical trials in DMO and wet macula degeneration that eyes with better baseline visual acuity have lower potential to gain letters [12–14]. In fact, the recent Protocol V study, demonstrated no advantage between observation, laser photocoagulation and intravitreal Aflibercept in patients with baseline visual acuity of 80 letters or more [15]. Both VISTA and VIVID recruited only patients with ≤73 letters at baseline and in our subgroup of patients with baseline visual acuity ≤70, the mean letter gain was approximately 3 letters less than the registration trials at +7.4. The results obtained in our ≤70 letters subgroup is consistent with that obtained in the French real-world APOLLON study of Aflibercept in diabetic macula oedema [16] (147 eyes, mean letter gain of +7.8 at 12 months in treatment-naïve cohort. It is slightly inferior to the outcomes of the Moorfield’s real world Aflibercept in DMO study [17] which reported +9.9 letter gain at 12 months. However, the Moorfield’s study had a much smaller sample size of 99 eyes, of which only 34% had good baseline visual acuity compared to 53.3% in the NWL cohort.

The mean reduction in CSFT at 12 months in this study was also lower compared to VISTA and VIVID. However, the NWL cohort started off with lower CSFT on average (mean baseline CST = 418) compared to 479 and 518 in VISTA and VIVID respectively and mean reduction in CSFT at 12 months increased to 142.34μm in the subgroup of NWL eyes with baseline CST ≥400μm.

The lower visual gains demonstrated in our study is also likely as a result of our wider inclusion criteria, as we did not perform fluorescein angiography to identify and exclude patients with significant macula ischaemia and we included patients with ocular comorbidities such as vitreomacular traction and epiretinal membrane, reflecting real-world routine practice, whilst the VIVID and VISTA trials excluded such patients and those with uncontrolled systemic diseases as would be expected. There are other significant differences in baseline patient demographics between the North West London cohort and VISTA and VIVID participants that likely contributed to lower visual gains. Over 80% of patients in VISTA and VIVID were Caucasian compared to 28% in the North West London cohort. Epidemiology data from the U.K and elsewhere indicates the prevalence of poor glycaemic control and sight threatening diabetic retinopathy is higher in Blacks and South Asians compared to Caucasians [18–21]. The mean HbA1c at baseline in VISTA and VIVID was 7.6, with 4.4% of participants categorised as proliferative diabetic retinopathy compared to a mean HbA1c of 8.3 in the NWL cohort and 15.9% categorised as proliferative diabetic retinopathy. Both higher HbA1C and presence of proliferative diabetic retinopathy are associated with poorer visual outcomes in eyes treated with intravitreal anti-VEGF for diabetic macula oedema [13,22]. As such, our results likely also reflect the suboptimal control of systemic disease in our population compared to the cohort recruited into VIVID and VISTA.^,^. In addition, whilst there is some evidence that ethnicity is a strong predictor of glycaemic control and albuminuria and individuals of South Asian and Afro-Carribean ethnicity may have suboptimal response to insulin [23,24], the influence of ethnicity as an independent predictor of response to intravitreal anti-VEGF agents has not been systematically investigated in the scientific literature.

There is evidence from post-hoc analysis of the VIVID and VISTA trials that there is incremental visual gain with each dose in the loading phase [25]. Therefore, we analysed the number of patients that completed their loading dose in our cohort and the reasons for missed doses. In our cohort, 45.3% did not complete their loading dose, of which 41.5% was the clinicians’ decision, 26.8% was due to lack of clinic capacity and 35% was due to missed appointment by the patient. This is common in the United Kingdom and a recent study evaluating patterns of loading phases of Aflibercept across 20 centres in the United Kingdom [26] found that the median number of injections delivered in the loading period was 3 and only 3 out of 20 centres managed to deliver 5 or more injections in the loading phase.

Additionally, in the ENDURANCE study [27], a 12 month extension of VISTA and VIVID, 30% of patients did not require further injections and visual acuity attained during VISTA and VIVID were maintained despite treatment on an ‘as-needed-basis’. However, on average the NWL cohort received approximately 2.5 injections less than was achieved in the clinical trials despite the treatment protocol in the 3 NWL hospitals following NICE guidelines. This reflects the difficulties of reviewing patients within the required interval requested by the clinician in healthcare organisations with finite resources as well as patient adherence to treatment. Our results are similar to other real-world studies investigating Aflibercept for diabetic macula oedema such as the APOLLON study (mean number of injections = 7.6) and the Moorfield’s study (mean number of injections = 6.92) and real world studies of intravitreal treatment of retinal disease in general [28,29].

Although we were unable to demonstrate a statistical difference between eyes completing the loading phase and those that did not in the NWL cohort, the study was not powered to detect this. As such, we postulate that the significant proportion of patients missing at least one loading dose and the lower number of injections delivered through the study period impacted visual gain at 12 months. We strongly recommend completion of the loading phase, particularly in cohorts with poor glycaemic control and severe diabetic retinopathy and measures that encourage adherence when commencing patients on Aflibercept for diabetic macula oedema.

Most patients in our study managed to complete at least 3 injections of the loading dose, so we further investigated whether response at 3 months was predictive of response at 12 months. Our analysis showed a clear positive correlation between both functional and anatomical response categories at 3 months and ongoing response at 12 months. In particular, only 6.5% of patients with limited visual gain went on to robust response by 12 months. Additionally, only 1 in 3 eyes with limited anatomical outcome demonstrated robust response by 12 months. Similar findings were described in post-hoc analysis of Protocol I [30,31], a multicentre clinical trial evaluating intravitreal ranibizumab for diabetic macula oedema. Our results suggest a change of therapy could be considered as early as 3 months in most patients with limited response although a small proportion will go on to develop delayed response with ongoing treatment. Factors that may identify late responders at baseline are still elusive.

Our study has limitations due to the retrospective nature, lack of strict visual acuity and OCT acquisition protocol as is the norm in clinical trials and the varied interpretation of need for retreatment that is expected in a multi-centre real-life setting. Nevertheless, our findings are more applicable to pressured healthcare systems serving a diverse population and highlight the challenges faced in real-world clinical settings when trying to deliver results obtained in idealised clinical trial settings. Our study highlights the broader variation in patient characteristics managed in real world settings and as such our outcomes are applicable to everyday clinical care and consistent with the efficacy of intravitreal anti-VEGF agents ‘in the wild’, outside strictly controlled clinical trial protocols.

## Conclusion

In summary, our study demonstrated that intravitreal Aflibercept was associated with improved functional and anatomical outcomes in the diverse North West London population, albeit lower than that demonstrated in the registration clinical trials. To our knowledge, this is the largest published dataset examining the efficacy of Aflibercept therapy delivered in a real-world setting in the United Kingdom and the first in such a diverse population. Intensive treatment in the loading phase is highly recommended in this cohort with poorer baseline glycaemic control and higher proportion of proliferative diabetic retinopathy compared to VISTA and VIVID. We demonstrate that poor compliance with treatment protocol and lack of clinic capacity remains a significant challenge that may have consequences for long-term visual outcome in the management of centre-involving diabetic macula oedema. Longer-acting agents with fewer loading doses remain an unmet need.

## Supporting information

S1 Dataset (XLSX)
