# Correction: Aflibercept for treatment-naïve diabetic macula oedema in a multi-ethnic population: Real-world outcomes from North West London

**DOI:** 10.1371/journal.pone.0257707

**Published:** 2021-09-16

**Authors:** Christiana Dinah, Arevik Ghulakhszian, Sing Yue Sim, Amal Minocha, Soroush Nokhostin, Esther Posner, Richard Cheong-Lee, Sheena George

## Notice of Republication

An incorrect version of S1 Dataset was published in error. The publisher apologizes for the error. This article was republished on September 7, 2021, to correct for this error. Please download this article again to view the correct version.
